# Tick-Borne Pathogen – Reversed and Conventional Discovery of Disease

**DOI:** 10.3389/fpubh.2014.00073

**Published:** 2014-07-07

**Authors:** Ellen Tijsse-Klasen, Marion P. G. Koopmans, Hein Sprong

**Affiliations:** ^1^Centre for Infectious Disease Control, National Institute for Public Health and the Environment (RIVM), Bilthoven, Netherlands; ^2^Erasmus Medical Center, Rotterdam, Netherlands

**Keywords:** tick-borne pathogens, public health, *Rickettsia*, *Neoehrlichia mikurensis*, *Ixodes ricinus*, *Borrelia miyamotoi*, emerging diseases

## Abstract

Molecular methods have increased the number of known microorganisms associated with ticks significantly. Some of these newly identified microorganisms are readily linked to human disease while others are yet unknown to cause human disease. The face of tick-borne disease discovery has changed with more diseases now being discovered in a “reversed way,” detecting disease cases only years after the tick-borne microorganism was first discovered. Compared to the conventional discovery of infectious diseases, reverse order discovery presents researchers with new challenges. Estimating public health risks of such agents is especially challenging, as case definitions and diagnostic procedures may initially be missing. We discuss the advantages and shortcomings of molecular methods, serology, and epidemiological studies that might be used to study some fundamental questions regarding newly identified tick-borne diseases. With increased tick-exposure and improved detection methods, more tick-borne microorganisms will be added to the list of pathogens causing disease in humans in the future.

## Introduction

Molecular methods, especially polymerase chain reaction (PCR), have brought huge changes to tick-borne disease research in the last two decades. A vast number of new microorganisms have been detected in ticks (1, 2), leading to an increase in reversed-disease discovery, where the microorganism is identified before its causal relationship with a disease is established (3–5). Several years can pass between the first detection of a microorganism in ticks and the first identification of a human case (4, 6). It is often unclear whether these novel tick-borne diseases were previously overlooked or if they were absent. Though molecular methods are not yet broadly used diagnostically, their increasing use in outpatient settings, as well as hospital settings, will improve the chance to identify novel tick-related microorganisms as causative agents of disease in future. There is a call for research on the growing lists of both new tick-related microorganisms with unknown pathogenicity and novel tick-borne pathogens for which the ecology, epidemiology, and full clinical picture are unknown, to elucidate their impact on public health.

## Discovery of Tick-Borne Diseases

### Reversed discovery of tick-borne diseases

Modern molecular methods enable the exploration of bacterial and viral communities in ticks without needing culturing. Since the 1990s, many studies have identified microorganisms in ticks that are distinct from known pathogens but cluster genetically with them. Frequently encountered bacteria in ticks with (initially) unknown pathogenicity are relatives of *Anaplasma*, *Bartonella*, *Ehrlichia*, and *Wolbachia*, and an increasing number of *Rickettsia* species (1, 2, 7). Now, we try to identify diseases caused by known microorganisms instead of looking for microorganisms causing known diseases (8, 9). This has led to an increase in pathogens about which only a few case reports exist and the disease burden – including clinical picture, severity, and incidence – is unclear.

Examples of reversed-disease discovery include *Neoehrlichia mikurensis*, *Borrelia miyamotoi*, and some *Rickettsia* species. *N. mikurensis* was discovered in 1999 in ticks by PCR and has since been reported in several countries (7, 10–12). Since 2010, serious diseases in immuno-compromised patients and mild disease in otherwise healthy individuals were associated with this bacterium (4, 13–16). Similarly, *B. miyamotoi* was found to cause disease in patients with febrile illness, Lyme, or anaplasmosis-like diseases years after it had been detected in ticks (5, 17–22). *Rickettsia helvetica* and *Rickettsia monacensis* had also first been identified in ticks before disease cases were linked to them (3, 23). These examples show that tick-borne infections can go unnoticed for various reasons. They might resemble known diseases or be overlooked due to non-specific symptoms. Furthermore, a lower disease incidence, due to a low exposure rate or due to a small susceptible population, can contribute to delayed discovery.

Ongoing developments in the field of next generation sequencing will deliver more sequence data of microorganisms in ticks (24). From this pool of microorganisms with unknown pathogenicity, more tick-borne pathogens could arise.

### Conventional discovery of tick-borne diseases

In contrast to reversed-disease discovery, conventional disease discovery starts with the identification of cases and the correlation with tick bites is recognized afterwards. This is facilitated if one or several of the following properties characterize the illness: serious disease course, temporal, or geographic clustering of cases or illness with characteristic symptoms (often a rash). These properties facilitate case definitions and epidemiological source tracing, thereby linking disease, tick-bite, and pathogen to a full etiological picture. Subsequently, the list of symptoms linked to the specific syndromes might be expanded, as demonstrated by the example of Lyme borreliosis.

In modern history, first correlations between tick bites and disease were observed around the turn of the last century (25). The first recognized tick-borne disease in humans was Rocky Mountain spotted fever (RMSF) (25), which drew attention since 1870s due to its high fatality rate, geographic and temporal clustering, and economic impact (26). Howard T. Ricketts identified the tick vector and the pathogen responsible for the disease (25, 26). Similarly to RMSF, tick-borne encephalitis (TBE) was identified in Russia due to the temporal clustering of cases that initiated an intensive search for the pathogen. The virus was isolated in 1937 (27). There have been severe cases with fatality rates between 1 and 40% depending on the subtype (28). Currently, the most commonly recognized tick-borne disease in humans is Lyme borreliosis, caused by members of the *B. burgdorferi* s.l. complex. Lyme borreliosis lacks the high mortality of RMSF and TBE but its typical rash, erythema migrans (EM) was recognized by Arvid Afzelius and other dermatologists in Europe in the early twentieth century (29, 30). There is a long list of differential diagnostics for other symptoms associated with Lyme borreliosis, including neurological, skeletomuscular, cardiac, and skin conditions (31). Therefore, the complete clinical spectrum of Lyme borreliosis was not recognized until 1970s, when an unusually high incidence of arthritis was observed in a small geographic area of the US (32). Tick-borne phleboviruses are the most recent pathogens identified following the conventional discovery route (33, 34). The first tick-borne phlebovirus was discovered in China after a small cluster of cases with thrombocytopenia and leukocytopenia provoked active surveillance for additional cases, identifying 285 patients. Cases were clustered in rural areas and a tick-borne etiology was soon suspected. The agent was then identified through metagenomic analysis of patient samples and later also detected in ticks (33). More examples for conventional discovery of tick-borne diseases are given in Table 1.

**Table 1:** **Selection of tick-borne diseases in humans and characteristics associated with their discovery**.

Disease	(Suspected) Pathogen	Disease first reported	Characteristic symptoms^a^	Temporal/geographic clusters	First isolated from	Diagnostic tests^b^	
Rocky Mountain spotted fever	*Rickettsia rickettsii*	1896	Yes	Yes	Humans	Yes	(25)
Relapsing fever	*Borrelia hermsii*, *B. duttonii*	1904	Yes	No	Humans	Yes	(35)
Mediterranean spotted fever	*R. conorii*	1910	Yes	No	Humans	Yes	(36)
Lyme (erythema migrans)	*B. burgdorferi* sensu lato	1912	Yes	No	Humans	Yes	(29)
Tick-borne encephalitis	TBE virus	1937	Yes	Yes	Humans	Yes	(37)
Human babesiosis	*Babesia microti*, *B. divergens*	1969	No	No	Livestock	Yes	(38, 39)
Lyme (whole syndrome)	*B. burgdorferi* sensu lato	1977	No	(Yes)	Humans	Yes	(32)
Anaplasmosis	*Anaplasma phagocytophilum*	1994	No	No	Livestock	Yes	(40)
Rickettsiosis	*R. helvetica*	1999	No	No	Ticks	No	(3)
Neoehrlichiosis	*Neoehrlichia mikurensis*	2010	No	No	Ticks	No	(4)
Lyme-like illness	*B. miyamotoi*	2011	No	No	Ticks	No	(5)

*^a^Characteristic symptoms do not need to occur in all patients with the infection*.

*^b^Commercially available diagnostic tests for the specific age*.

### From non-pathogenic to established pathogen

Microorganisms detected in ticks can have different implications for human health. Some have not been shown to cause disease in humans while others are established human pathogens. Non-pathogenic microorganisms detected by molecular methods in ticks include tick endosymbionts, commensal bacteria, and residual DNA from earlier blood meals (24, 41, 42). Established pathogens include agents such as *R. rickettsii*, TBE, and *B. burgdorferi* s.l., which are well described and known to cause disease.

When tick-borne diseases are identified following the reversed course of disease discovery, they progress from the category of non-pathogens to pathogenic microorganisms. However, as information about ecology, epidemiology, and clinical picture are initially lacking, further research is necessary to confirm pathogenicity, incidence, and geographic distribution. Currently, a number of novel tick-borne microorganisms fall in this category, including *R. helvetica*, *N. mikurensis*, and *B. miyamotoi* (34). The public health relevance of such suspected tick-borne pathogens is unknown and should be one of the key objectives of further studies. Some of these novel tick-borne pathogens might be involved in yet unexplained disease following tick bites or acute or chronic inflammation without known cause. To assess actual health impact and relevance, a causal relationship needs to be confirmed by a strong line of evidence, for example, following Koch’s postulates (Box 1). Case definitions have to be established and prevalence of disease needs to be estimated.

Box 1 **Tick-borne diseases and Koch’s postulates.**

Providing evidence for a causal relationship between a tick-borne microorganism and a certain disease can be challenging. About 120 years ago, Jakob Henle and his student Robert Koch formulated three postulates to help prove a causal relationship between an infectious agent and a disease. If the following points are met, it can be concluded that the parasite has a causal relationship with the disease in question [freely translated from Ref. (43)]:

1. The parasite is found in every case of the disease in question, in circumstances under which it can account for pathological changes and the clinical course of the disease.
2. The parasite is not found in any other disease as a fortuitous and non-pathogenic parasite.
3. After complete isolation from the body and grown repeatedly in pure cultures, the parasite is again able to produce the disease.

To meet the postulates, the agent must be culturable and cause the same disease invariably in a new host (human or experimental animal). However, many pathogens, including some novel tick-borne microorganisms, cannot fulfill these premises or lack suitable animal models (44). Koch was the first to identify asymptomatic carriers of a pathogen and was thus aware of these limitations (43, 45) that also restrict the applicability of his original postulates for tick-borne pathogens. However, several alternatives have been formulated (46–49). The postulates of Fredericks and Relman (48) rely on sequence-based detection of pathogen DNA in tissue samples. Making no absolute statements, they emphasize the importance of higher amounts of DNA and higher incidence of DNA detection in cases compared to controls, while the DNA load should fall or rise with disease resolution or recurrence. Evans formulated several premises that should be met, including epidemiological measures (e.g., higher disease incidence in those carrying an organism), host response (e.g., serology), and effectiveness of preventative measures (46). Other authors acknowledge the value of direct visualization of infectious agent, strain differences, serology, epidemiology and, especially, combinations of these (46, 47). A conclusive line of evidence for the causal role of an infectious agent in a specific disease supported by classical or alternative postulates would be ideal, but might not be realistic for some of the novel tick-borne pathogens in the near future.

## Methods for the Discovery of Novel Tick-Borne Pathogens and the Estimation of Their Public Health Impact

Estimating the disease burden of novel tick-borne diseases and microorganisms with unknown pathogenicity should be the focus of research in this area. This requires studies on many levels. Information about the ecology of novel tick-borne diseases, including the vector, natural cycle, and reservoirs of the microorganism can help to identify high-risk regions and populations. In the long run, this information could also be helpful in identifying counteractions such as culling reservoir animals (if compatible with nature conservation efforts) or other ways to reduce tick density. Data about the epidemiology will help to identify peak periods, estimate disease incidence, and the overall public health impact of novel tick-borne pathogens (50). Finally yet importantly, knowledge about symptoms associated with novel tick-borne pathogens and knowledge about risk factors provide health practitioners with tools to identify potential cases. Identifying cases is important to request appropriate diagnostic tests and initiate appropriate treatment and request appropriate diagnostic tests. This in turn might help epidemiological data collection.

### Identification of potential pathogens in ticks and possible pitfalls

Microorganisms in ticks are most commonly detected and identified by PCR and direct sequencing. 16S rDNA library and next generation sequencing methods have also been used (24, 51–53). With decreasing costs in the future, the latter will probably gain importance and open new doors to microbial discovery. Sequencing several genes of a novel tick-related microorganism can also give a preliminary estimation of the microorganism’s pathogenic potential. Some genes, such as the surface protein OspC of *B. burgdorferi* sl., might be directly linked to pathogenicity (54). However, with novel microorganisms, such associations are usually unknown. A comparison of the microorganisms’ overall genetic background with that of known pathogens might help. *Rickettsia* species, for example, are plentiful in invertebrates of which only a fraction is found in vector species (55). A first evaluation based on several gene sequences can help to determine whether a novel *Rickettsial* species clusters in one of two known groups that contain human pathogens: the typhus and the spotted fever group. A *Rickettsia* species not belonging to one of these pathogen-containing groups has therefore a lower chance to be pathogenic. More advanced predictions based on whole genome sequencing are also underway and might assist in the identification of tick-borne pathogens in the future (56). However, it should be noted that molecular techniques have weaknesses, including the inability to distinguish living and dead cells and the risk of contamination or PCR artifacts from various sources. Although not yet shown for ticks, in some cases the detection of a single gene might also be due to horizontal gene transfer (57). One source of misleading PCR results was recently discovered. Eggs of a parasitic wasp, *Ixodiphagus hookeri*, can be embedded in ticks collected in the field. The eggs contain *Wolbachia* but more bacteria or viruses might be present in them and lead to misleading PCR results (58, 59).

### Knowledge about ecological factors can guide search for disease

**Figure 1:**
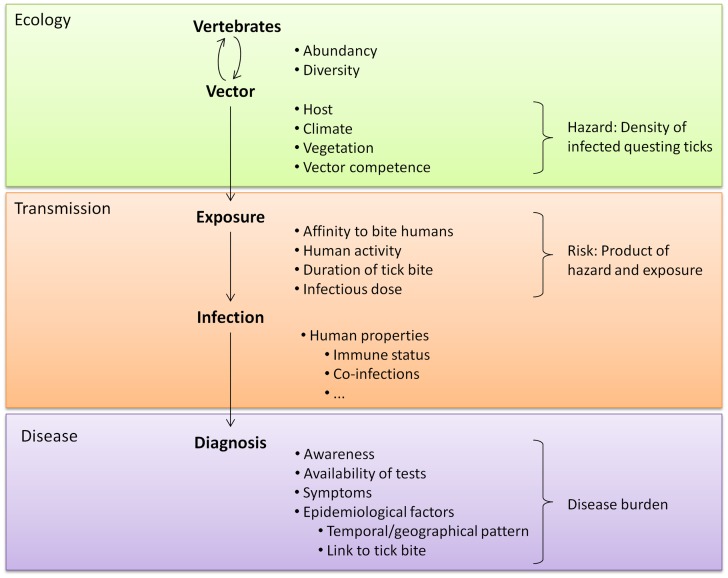
**A tick-related microorganism must take various steps to cause disease**. Each step is influenced by many factors, including the characteristics of the microorganism. These characteristics affect every step of transmission and are therefore not listed separately.

Studying novel tick-borne pathogens and the diseases they cause can be facilitated by knowledge of the microorganism’s ecology. Tick species that can act as vectors include generalist species that readily bite humans (e.g., *I*. *ricinus* and *Amblyomma americanum*) but also opportunistic species that prefer other vertebrate hosts (e.g., *Rhipicephalus sanguineus*) (60). Environmental factors can influence tick densities and the prevalence of tick-borne microorganisms and can thus influence exposure risks for humans (61–67) (Figure 1). Understanding the relationships between ecological factors and the prevalence of tick-borne pathogens as well as mapping densities of infected ticks can help to identify high-risk areas for human exposure. Furthermore, knowledge about natural cycles of vectors, hosts and pathogens might help to predict seasonal variations in pathogen prevalence (50). Even for pathogens transmitted by the same tick species, peak periods of disease cases can vary because disease incidence does not only depend on the questing activity of ticks. First of all, different tick-borne diseases can be transmitted by different tick stages (e.g., *Rickettsia* vs. *B. burgdorferi*) and these differ in their main questing period. Secondly, the infection rate of ticks with various pathogens can underlay different seasonal variations as was shown by Coipan et al. (50). However, if no data are available about seasonality of novel tick-borne pathogens, peaks in established tick-borne diseases vectored by the same tick species could indicate similar seasonal patterns of novel pathogens (50). Aligning the start and location of epidemiological studies and sampling periods of serological studies with such high-risk areas and high-risk periods could improve the chance to identify cases of a novel tick-borne disease.

### Serology for identification of exposure and cases

Serological methods have a wide diagnostic window, as antibodies to a pathogen may persist for months or even years. This makes these assays valuable to investigate past exposure to tick-borne pathogens. Advantages of serology include the ease of obtaining samples and ability to detect current infections by sero-conversion. Serology can be used to investigate infection with tick-borne pathogens in high-risk populations or those showing signs of disease with unknown cause. Examples of the latter are the investigation into possible rickettsial origins of chronic illness in Australian patients and of liver dysfunctions in Spanish patients (68, 69). In these studies, patient groups had a higher seroprevalence for rickettsial antigens than controls. Cochez and coworkers screened paired sera of 322 patients with suspicion of tick-borne infections for the presence of *Anaplasma phagocytophilum* antigen and found evidence of infection in about a third of the patients (70). Such investigations do not prove causal relationships of disease with a specific tick-borne pathogen but could be the key to identifying certain clinical entities.

Highly specific serology would facilitate this kind of study. However, for many tick-borne diseases, serological assays need improvement as they lack sensitivity or the specificity to differentiate between species or genotypes. For some of the newest tick-borne diseases, no serological assays are available at all. Fast development of serological assays often relies on culture for antigen production. In cases where cultures are not available, production of recombinant proteins or synthetic peptides offer an alternative (22, 71). Both require genetic information on potentially antigenic proteins. A further bottleneck in the development of serological assays is the availability of suitable samples for validation. Especially if a microorganism is suspected to be involved in disease but positively identified cases are scarce or lacking, the availability of well-defined sera for validation purposes is limited. Disregarding the type of antigen used, the inability to distinguish asymptomatic infections from disease and lack of immune response under some circumstances limit the use of serology, as it can only be a measure for infection risk rather than disease risk (72, 73). For these reasons, the use of serology in novel tick-borne disease research is limited but can have increased value when combined with other methodologies such as molecular detection of pathogens or large epidemiological studies linking sero-conversion with clinical manifestations of disease.

### Molecular methods for identification of infections

The development of serology for novel pathogens generally takes time, while PCR is often already available or can be set up quickly. Molecular methods enable testing for tick-borne pathogens for which no serological assays exist. The limitation of PCR on patient material, besides contamination risks, lies in the availability of suitable material. Tissue tropisms differ for different pathogens or clinical presentations (74). Therefore, the choice of the tissue to be tested is crucial for success, requiring a certain degree of knowledge about the pathogenesis of a microorganism. This can be derived from previous case reports, wildlife and animal studies, or tissue tropisms of related pathogens. Samples that might be available for testing include skin biopsies, tissues removed during medically required surgery, cerebrospinal, synovial fluids, and blood samples. Blood samples, being so readily available, will often be the first though potentially not always the best choice to test for tick-borne pathogens and have been successfully used (5, 75). Skin biopsies have been useful in the diagnosis of rickettsiosis, or for research purposes on various rashes, including EM (76, 77). Novel tick-borne diseases have not yet been identified by PCR on skin samples but this may change in future.

Detection of a microorganisms’ DNA in a single patient does not prove a causal relationship. The microorganism might not be the causative agent of the observed disease but a mere asymptomatic co-infection. However, using molecular techniques on larger case numbers and analyzing the data according to specified parameters [e.g., with adapted Koch’s postulates (48)] could help support causal relationships and formulate case definitions.

### Epidemiological studies to define disease incidence and identify cases

Epidemiological studies to link tick bites with health outcome vary in their design from retrospective to prospective and from case–control to cohort studies. Prospective cohort studies have been performed to find associations between tick-borne pathogens and adverse health effects or serological response (78–82). Prospective cohort studies combine high precision-of-risk estimations, the ability to study several outcomes at once, and opportunity to include (molecular) data collected from ticks, if available. While past studies with 250–400 tick-bitten participants detected some Lyme borreliosis cases, they failed to identify cases caused by other pathogens that might have a lower incidence (either in due to lower prevalence in ticks or lower infectivity) (81, 82). This highlights the drawbacks: success of cohort studies depends on the size of the study population, the fraction of exposed individuals, and the frequency of the expected outcome in exposed individuals. Consequently, cohort studies are less suitable for uncommon pathogens or those with a low pathogenicity, unless the cohort is very large. Studies focusing on syndromic surveillance and diagnosis of high-risk patients, ideally coupled with case–control studies to identify causal factors, would be more suitable to detect rare tick-borne diseases. Identifying a patient group with symptoms or laboratory findings matching earlier case descriptions increases the chance of detecting novel tick-borne pathogens in patients (21). Focusing on patients with tick-exposure history or on areas with a high prevalence of the pathogen will further increase the chance of identifying cases. Identifying individual cases is crucial to answering some questions concerning novel tick-borne diseases, such as the full clinical picture and risk factors. In contrast to individual case studies, epidemiological studies can supply valuable data to help risk estimation and disease burden of newly identified tick-borne diseases.

## Summary

Tick-borne disease research has changed greatly since the age of molecular detection methods. An increasing number of novel tick-related microorganisms are being identified and this evolution will continue in future due to the increasing availability of new sequencing methods. Isolated cases of human diseases caused by novel tick-borne microorganisms can suggest that a microorganism is pathogenic but they do not provide sufficient proof of a causal relationship. A causal relationship of a novel pathogen with a disease would be supported by the use of Koch’s postulates (Box 1).

The rigid criteria used in the original postulates might not be suitable, though, in which case modern adaptations of the postulates can demonstrate a causal role (48, 49). Such alternative postulates can rely on serology, molecular diagnostics, or epidemiology. Novel tick-borne pathogens could play a role in diseases with currently unknown etiology, such as chronic fatigue, skeletomuscular, and neurological symptoms (5, 20). They might also explain treatment-resistant symptoms in patients diagnosed with other tick-borne diseases (20). Knowledge about a pathogen’s ecology could be used to guide such studies by identifying high-risk areas and populations. Ecological knowledge might also be useful to educate the public and take measures to reduce the density of infected ticks. Efforts to increase awareness among medical health professionals, providing diagnostic tools (case definition, serology, PCR, etc.) and recommending effective treatment options will further help to diagnose and treat cases.

The incidence of some tick-borne diseases showed an increase or fluctuations throughout recent decades due to various factors, mainly associated with increased tick-exposure (82–84). This upward trend might extend to newly identified tick-borne diseases as well, as these also depend on tick-exposure. People with co-morbidities are more likely to develop (severe) disease following infection with tick-borne pathogens and this sensitive group is growing due to current medical and sociological developments (4, 21). It is likely that even in the healthy population many cases caused by novel tick-borne pathogens go unnoticed. The real number of cases could therefore be significantly higher than currently apparent. The actual incidence needs to be determined to help estimate public health impact.

## Conflict of Interest Statement

The authors declare that the research was conducted in the absence of any commercial or financial relationships that could be construed as a potential conflict of interest.
